# Supplementary material for: A comparison between scalp- and source-reconstructed EEG networks
Source: Sci Rep. 2018 Aug 16;8:12269. doi: 10.1038/s41598-018-30869-w (PMC6095906; doi:10.1038/s41598-018-30869-w)
Supplement: Supplementary file 1 — Supplementary information [file 41598_2018_30869_MOESM1_ESM.docx]

**Supplementary information.**

**A comparison between scalp- and source-reconstructed EEG networks**

Margherita Lai^1^, Matteo Demuru^2^, Arjan Hillebrand^2^, Matteo Fraschini^1,*^

^1^ Department of Electrical and Electronic Engineering, University of Cagliari, Piazza D'armi, Cagliari, I-09123, Italy

^2^ Department of Clinical Neurophysiology and MEG Center, VU University Medical Centre, Amsterdam, The Netherlands

**1. Correlations with reduced network size.**

In order to estimate the effect of the small difference in network size (64 nodes at scalp level versus 68 nodes at source-level) on the correlations as reported in the main manuscript, we here report results obtained using a subset of nodes for the source analysis. In particular, we omitted four nodes from original atlas (right and left parahippocampal and lingual ROIs), thus obtaining a network with 64 nodes at the source-level (and using the original 64 nodes at the scalp-level).

Here, we report the analysis for the MST leaf fraction based on the corrected AEC, which provided the higher correlations in respect to the other FC metrics. As can be seen in Figure S1, the correlation between the leaf fraction obtain for the scalp- and source-level MSTs is comparable for the case where the network size was equal (rho= .355) and when the network sizes differed (rho= .345), 64 electrodes versus 68 ROIs. The impact of the small difference in network size was thus negligible, since both the direction and the strength of associations (as measured by Spearman correlations) were comparable (difference = -0.01 [-0.03 0.01]).

*
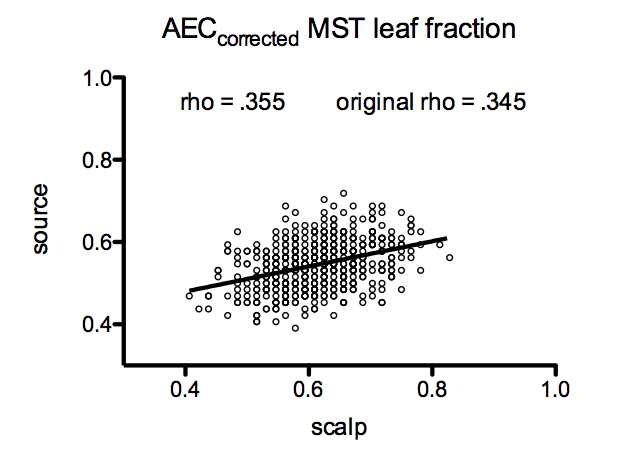
*

Figure S1. Correlation between scalp- and source-level analysis for MST leaf fraction based on AEC_corrected_.

**2. Replication using sLoreta**

In order to examine the potential effect of other inverse methods, we have replicated the original analysis that was done with the wMNE, using the sLoreta approach. This, as reported in Figure S2, provided results in terms of correlations between scalp- and source–level results comparable with those obtained using wMNE.


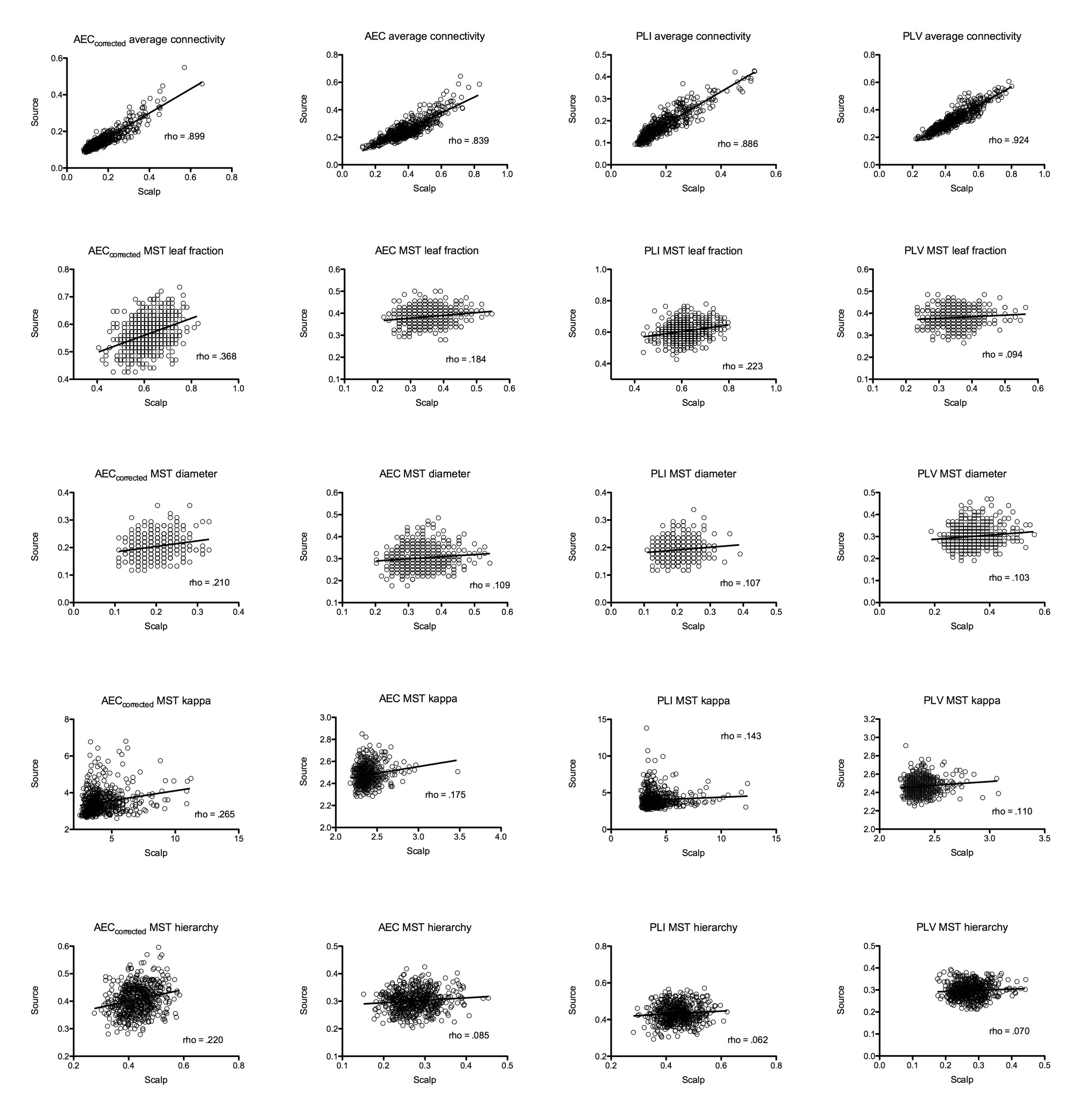


Figure S2: Scatterplots of scalp- and source-based measures of FC and network topology using sLoreta. The strength of the correlation is reported as rho value.

**3. Replication using of efficiency cost optimization (ECO) as a thresholding approach**

In this section we show the results obtained using a new thresholding approach, the efficiency cost optimization (ECO), recently introduced by De Vico Fallani et al ^1^. We found that using ECO supports our previous findings (obtained by using the MST), where AEC_corrected_ and PLI show (see the Figure below) again a higher correlation between the two domains, and very low correlations for AEC and PLV. In this case we show the results using the global efficiency as network measure. The results derived by using the ECO approach shows slightly higher correlations in comparison with those obtained using the MST for both AEC_corrected_ and PLI.

*
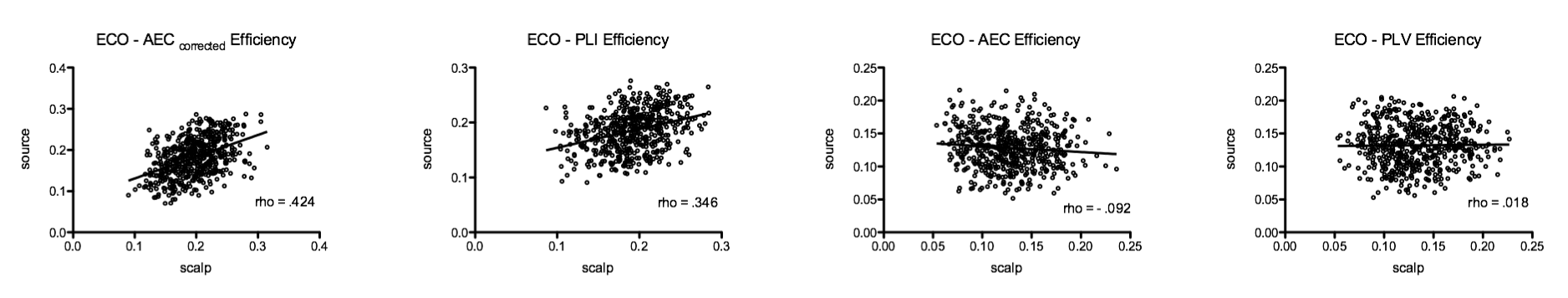
*


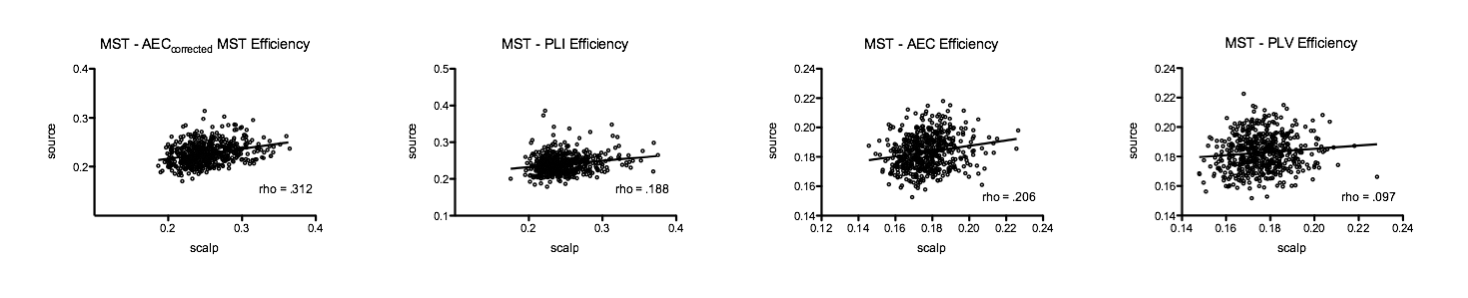


Figure S3. Correlations between scalp- and source-level analysis global efficiency for each connectivity metrics computed the two different thresholding approaches, ECO (upper row) and MST (bottom row).

**4. Replication using other frequency bands**

In order to exclude that our findings were only due to the investigated frequency content, we have analysed the MST leaf fraction, a measure that characterizes the global topology of the network, and which was shown to give a relatively high correlation between scalp- and source-level analysis for AEC_corrected_, also for the theta and beta bands. In Figure S4 we show the correlations between scalp and source estimated for theta (4-8 Hz) and beta (13-30 Hz) bands, giving similar correlation as for the alpha band (rho = .346).

*
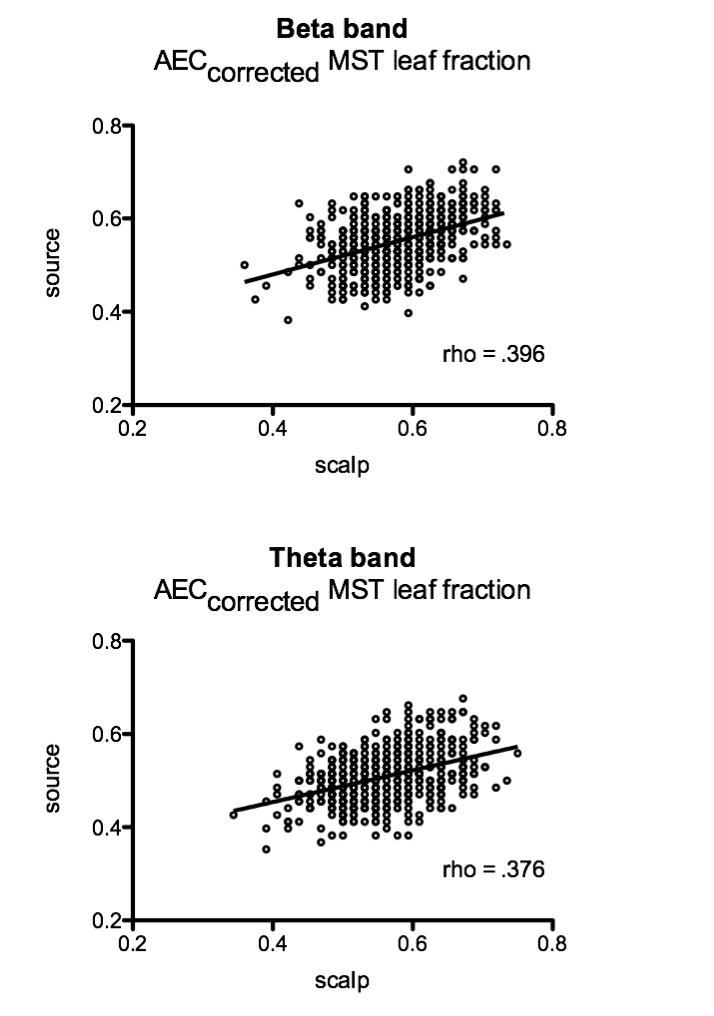
*

Figure S4. Correlations between scalp- and source-level analysis MST leaf fraction for AEC_corrected_ for beta and theta frequency bands.

1. De Vico Fallani, F., Latora, V. & Chavez, M. A Topological Criterion for Filtering Information in Complex Brain Networks. *PLOS Comput. Biol.* **13,** e1005305 (2017).
